# Midkine, a Potential Link between Obesity and Insulin Resistance

**DOI:** 10.1371/journal.pone.0088299

**Published:** 2014-02-07

**Authors:** Nengguang Fan, Haiyan Sun, Yifei Wang, Lijuan Zhang, Zhenhua Xia, Liang Peng, Yanqiang Hou, Weiqin Shen, Rui Liu, Yongde Peng

**Affiliations:** 1 Department of Endocrinology, Shanghai First People’s Hospital, Shanghai Jiao Tong University, Shanghai, China; 2 Department of Endocrinology, Shanghai Songjiang Center Hospital, Shanghai, China; 3 Department of Laboratory Medicine, Shanghai Songjiang Center Hospital, Shanghai, China; Universidad Pablo de Olavide, Centro Andaluz de Biología del Desarrollo-CSIC, Spain

## Abstract

Obesity is associated with increased production of inflammatory mediators in adipose tissue, which contributes to chronic inflammation and insulin resistance. Midkine (MK) is a heparin-binding growth factor with potent proinflammatory activities. We aimed to test whether MK is associated with obesity and has a role in insulin resistance. It was found that MK was expressed in adipocytes and regulated by inflammatory modulators (TNF-α and rosiglitazone). In addition, a significant increase in MK levels was observed in adipose tissue of obese ob/ob mice as well as in serum of overweight/obese subjects when compared with their respective controls. *In vitro* studies further revealed that MK impaired insulin signaling in 3T3-L1 adipocytes, as indicated by reduced phosphorylation of Akt and IRS-1 and decreased translocation of glucose transporter 4 (GLUT4) to the plasma membrane in response to insulin stimulation. Moreover, MK activated the STAT3-suppressor of cytokine signaling 3 (SOCS3) pathway in adipocytes. Thus, MK is a novel adipocyte-secreted factor associated with obesity and inhibition of insulin signaling in adipocytes. It may provide a potential link between obesity and insulin resistance.

## Introduction

Obesity has become a global epidemic that is closely associated with the development of insulin resistance, type 2 diabetes and cardiovascular diseases [1], [2]. Initially viewed as a major site for energy storage, adipose tissue has recently been identified as an important endocrine and immune organ [3], [4]. It secretes a variety of bioactive molecules, including adiponectin, leptin, and various inflammatory mediators (e.g., TNF-α, IL-6 and MCP-1), which are collectively termed as adipokines [3], [4]. Obesity leads to a dramatically changed secretory profile of adipose tissue, characterized by increased production of proinflammatory cytokines, such as TNF-α, IL-1β and IL-6 [5], [6]. These cytokines exert direct actions on adipocytes and other insulin target cells, inducing chronic inflammation and insulin resistance [5], [6]. To date, many novel adipokines with proinflammatory properties have been identified and linked to obesity-induced inflammation and insulin resistance [7].

Midkine (MK), also known as neurite growth-promoting factor 2, is a 13-kDa heparin-binding growth factor with pleiotropic activities [8]. It was originally identified as a retinoic acid-inducible molecule in mouse embryonic carcinoma cells, and is expressed in mouse embryos at mid-gestation [9]. Structurally, MK shares 50% sequence identity with pleiotrophin, both of which are composed of two domains (N- and C-domain) [9], [10]. It has been shown that MK promotes cell proliferation, differentiation, survival and migration, and is involved in a variety of biological processes, including neuronal development, angiogenesis and oncogenesis [10]–[13]. In addition, growing evidence has indicated a key role of MK in inflammation [14]. It promotes chemotaxis of neutrophils and macrophages and suppresses expansion of regulatory T cells [15]–[17]. Accordingly, MK-deficient mice were protected against antibody-induced rheumatoid arthritis, neointima formation after vascular injury, and experimental autoimmune encephalomyelitis, associated with decreased inflammatory cell infiltration and enhanced regulatory T cell expansion [16]–[18]. Clinically, patients with inflammatory diseases including rheumatoid arthritis, ulcerative colitis and Crohn’s disease had increased blood MK compared with control subjects [18]–[20]. Together, MK appears to be a mediator implicated in many inflammatory processes and diseases. However, the relationship between MK and obesity, a state of chronic inflammation, is unclear.

Indeed, MK is synthesized and secreted by adipocytes [21]. During in vitro adipogenesis of 3T3-L1 preadipocytes, MK expression was markedly increased after initiation of differentiation. It exerted an essential role in the mitotic clonal expansion of 3T3-L1 preadipocytes [21], in line with its mitogenic effects on other cell types [22], [23]. These in vitro findings seem to have their clinical relevance. Compared with control subjects, obese and diabetic children and adolescents had significantly higher levels of serum MK [24]. However, the relationship between MK and obesity and the role of MK in mature adipocytes remain to be further determined.

In the present study, we initially assessed MK expression levels in 3T3-L1 adipocytes and its regulation by inflammatory modulators. Then, we investigated the association between MK and obesity by examining MK levels in adipose tissue of mice and in serum of humans. Furthermore, in vitro experiments were performed to investigate the impact of MK on insulin signaling and GLUT4 translocation in 3T3-L1 adipocytes. Finally, the proinflammatory effects of MK on adipocytes were determined.

## Materials and Methods

### Ethics Statement

All research involving human participants was approved by the Institutional Review Board of Shanghai First People’s Hospital affiliated to Shanghai Jiao Tong University School of Medicine, and performed in accordance with the principle of the Helsinki Declaration II. Written informed consent was obtained from all subjects. Animal procedures were approved by the Committee on the Ethics of Animal Experiments of Shanghai Jiao Tong University and were carried out in strict accordance with the recommendations in the Guide for the Care and Use of Laboratory Animals of Shanghai Jiao Tong University. All operations were performed under sodium pentobarbital anesthesia, and all efforts were made to minimize suffering.

### Chemicals and Reagents

Dulbecco’s modified Eagle’s medium (DMEM) was purchased from Hyclone (Beijing, China). Fetal bovine serum (FBS) and penicillin-streptomycin were from Gibco (Carlsbad, CA). Isobutylmethylxanthine, dexamethasone, insulin and rosiglitazone were from Sigma (St. Louis, MO). Recombinant mouse MK was obtained from Pepro Tech (Rocky Hill, NJ) and the endotoxin level was below 1.0 EU per 1 µg of the protein by the LAL method. Human TNF-α was also from Pepro Tech (Rocky Hill, NJ). Specific antibodies against STAT3, phospho-STAT3 (Tyr705), phospho-p65 (Ser536), Akt, phospho-Akt (Ser473), GLUT4 and GAPDH were purchased from Cell signaling Technology (Beverly, MA). Antibody against MK was from Santa Cruz Biotechnology (Santa Cruz, CA). Phospho-IRS1 (Tyr612) antibody was from Abcam (Cambridge, MA). Na/K ATPase α-1 antibody was obtained from Novus Biologicals (Littleton, CO). Horseradish peroxidase-conjugated antibodies against rabbit or goat IgG were from Jackson Laboratories (West Grove, PA).

### Subjects

A total of 206 individuals who consecutively visited the Medical Examination Center of Shanghai First People’s Hospital for routine health check-ups were invited and 165 individuals agreed to attend our study. After excluding 30 ineligible subjects with diabetes, acute or chronic infectious diseases, autoimmune diseases, heart failure, hepatic or renal diseases, 135 individuals were included in our final analysis. Based on body mass index (BMI), the subjects were divided into two groups: normal weight (NW; BMI <25 kg/m^2^, n = 84) and overweight/obese subjects (OW/OB; BMI ≥25 kg/m^2^, n = 51).

### Anthropometric and Biochemical Measurements

All subjects were assessed after overnight fasting for at least 10 h. Body weight, height, systolic blood pressure (SBP) and diastolic blood pressure (DBP) were measured by an experienced physician. BMI was calculated as body weight in kilograms divided by body height squared in meters. Two 5-ml blood samples were collected from the cubital vein by one experienced nurse. Fasting blood glucose (FBG), triglycerides (TG), total cholesterol (TC), low-density lipoprotein cholesterol (LDL-C), and high-density lipoprotein cholesterol (HDL-C) were measured using an autoanalyzer (Beckman, Palo Alto, CA). Serum MK levels were determined with a commercially available enzyme-linked immunosorbent assay (ELISA) kit (DuoSet, R&D Systems, Minneapolis, MN). The linear range of the assay was 78.1–5000 pg/ml.

### Animals

Male C57BL/6J leptin-deficient (ob/ob) mice and their lean littermates (6 weeks of age; n = 4 per group) were purchased from the Model Animal Research Center of Nanjing University (Nanjing, China). Mice were housed in a pathogen-free barrier facility with a 12 h light/12 h dark cycle, and given free access to water and standard chow diet (Slaccss, Shanghai) containing 20% protein and 5% fat (w/w). At 16 weeks of age, all mice were sacrificed under sodium pentobarbital anesthesia. Then, epididymal adipose tissues of the mice were immediately desected and fixed in 4% paraformaldehyde at 4°C, or snap-frozen in liquid nitrogen and stored at −80°C until use.

### Cell Culture and Treatment

3T3-L1 preadipocytes and RAW264.7 macrophages were obtained from American Type Culture Collection (Rockville, MD) and maintained in DMEM supplemented with 10% FBS, 100 U/ml penicillin and 100 µg/ml streptomycin in a 5% CO2 humidified atmosphere at 37°C. Differentiation of 3T3-L1 preadipocytes was performed as described previously [25]. Briefly, 2 days postconfluence (defined as D0), cells were exposed to differentiation medium containing 0.5 mM isobutylmethylxanthine, 1 µM dexamethasone, 1.67 µM insulin (MDI) and 10% FBS. After 48 h of incubation (D2), the medium was replaced with DMEM containing 10% FBS and 1.67 µM insulin. On D4, the cells were switched to DMEM containing 10% FBS and refed every other day for the following 4–6 days until the cells were fully differentiated. Typically, more than 90% of the 3T3-L1 cells showed accumulation of multiple lipid droplets as determined by staining with Oil Red O. Before each treatment, fully differentiated 3T3-L1 adipocytes were serum starved in DMEM containing 0.25% FBS for 16 h.

To explore the effects of TNF-α and rosiglitazone on the expression of MK, serum-starved 3T3-L1 adipocytes were treated with or without TNF-α (20 ng/ml) in the presence or absence of rosiglitazone (1 µM) for 24 h. RNA and protein were extracted to evaluate the relative expression of MK mRNA by RT-PCR, and protein by western blot. To examine the role of MK on insulin signaling, serum-starved 3T3-L1 adipocytes were exposed to recombinant mouse MK (100 and 200 ng/ml) or vehicle for 24 h, followed by stimulation with 100 nM insulin for 10 min. Subsequently, phosphorylation of Akt (Ser473) and IRS-1 (Tyr612) were assessed by western blot analysis. When assessing the impact of MK on GLUT4 translocation, 3T3-L1 adipocytes were treated with MK (200 ng/ml) for 24 h, followed by insulin (100 nM) stimulation for 30 min. Plasma membrane proteins were isolated and subjected to western blot. To further determine the potential mechanisms underlying the effects of MK on insulin signaling, differentiated 3T3-L1 adipocytes were treated with recombinant MK (100 ng/ml) for various time periods. Phosphorylated (Tyr705) and total STAT3 protein levels were assessed by western blot analysis. Moreover, SOCS3 mRNA expression was evaluated in 3T3-L1 adipocytes treated with increasing dose of recombinant mouse MK (0, 50, 100 and 200 ng/ml) for 16 h. The cellular experiments were repeated at least 3 times.

### RNA Preparation and Quantitative Real-time PCR Analysis

Total RNA was extracted from adipose tissues or cells with TRIzol Reagent (Invitrogen, Carlsbad, CA) according to the manufacturer’s instructions. Next, 1 µg of total RNA was reverse-transcribed into first-strand cDNA using the Reverse Transcription system (Promega, Madison, WI). Quantitative real-time PCR was then performed in duplicate using the SYBR premix Ex Taq kit (TaKaRa, Dalian, China) on a DNA Engine Opticon 2 Real-Time PCR Detection System (Bio-Rad, Hercules, CA). Reaction conditions were 95°C for 2 min, and then 40 cycles of 95°C for 15 s/60°C for 30 s. The primer sequences are listed in Table 1. Gene expression was normalized to β-actin using the ΔΔct method.

**Table 1:** Primer sequences for real-time PCR.

Genes	Sense	Anti-sense
Midkine	TGGAGCCGACTGCAAATACAA	GGCTTAGTCACGCGGATGG
SOCS3	ATGGTCACCCACAGCAAGTTT	TCCAGTAGAATCCGCTCTCCT
IL-6	GAGGATACCACTCCCAACAGACC	AAGTGCATCATCGTTGTTCATACA
MCP-1	CTTCTGGGCCTGCTGTTCA	CCAGCCTACTCATTGGGATCA
β-actin	GGCTGTATTCCCCTCCATCG	CCAGTTGGTAACAATGCCATGT

### Western Blot Analysis

For whole cell protein extraction, adipose tissues or cells were lysed in RIPA buffer (50 mM Tris-HCl, pH 7.4, 150 mM NaCl, 1% NP-40, 0.5% sodium deoxycholate, 0.1% SDS) containing protease and phosphatase inhibitors (5 mM EDTA, 1 mM PMSF, 1 mM sodium orthovanadate) for 30 min on ice. After centrifugation, the supernatants were collected and protein concentrations were determined by the BCA protein assay (Pierce, Rockford, IL). For plasma membrane protein isolation, the Pierce Cell Surface Protein Isolation Kit was used according to the manufacturer’s instructions (Pierce, Rockford, IL). Equal amounts of protein from each sample were electrophoresed on 12% SDS-PAGE gels and then transferred to polyvinylidene difluoride membranes (Millipore, Bedford, MA). The membranes were blocked with 5% skim milk in TBS containing 0.1% Tween-20 for 1 h at room temperature, and then incubated with different primary antibodies overnight at 4°C. After washing and incubating with HRP-conjugated secondary antibodies for 1 h at room temperature, immunoreactive proteins were visualized using SuperSignal Pico ECL reagent (Pierce, Rockford, IL) and exposed to film. To reprobe with different antibodies, the membranes were stripped in stripping buffer containing 62.5 mM Tris·HCl, PH 6.8, 2% SDS, and 100 mM β-mercaptoethanol at 50°C for 20–30 min with shaking.

### Immunohistochemical Analysis

Adipose tissues fixed in 4% paraformaldehyde were embedded in paraffin and sectioned to a thickness of 5 µm. The sections were then deparaffinized in xylene and endogenous peroxidase activity was depleted with 0.3% hydrogen peroxide for 30 min at room temperature. For immunostaining of MK, the sections were first blocked with phosphate-buffered saline (PBS) containing 5% normal goat serum for 60 min at room temperature, followed by incubation with goat anti-MK antibody overnight at 4°C. The sections were washed three times with PBS and then incubated with HRP-conjugated rabbit anti-goat secondary antibody for 1 h at room temperature. After three washes with PBS, the sections were incubated with 0.1% diaminobenzidine (DAB) solution for 5–10 min. The nuclei were counterstained with hematoxylin for 5 min. Finally, images were acquired on a Zeiss microscope fitted with an Axiocam MRc camera and using Axiovision software (Carl Zeiss, Thornwood, NY).

### Statistical Analysis

Data are presented as means ± SE unless otherwise stated. Non-normally distributed data were logarithmically transformed before analysis. Comparisons between groups were carried out using unpaired Student’s t-test (for comparisons between two groups) or one-way ANOVA with Bonferroni post hoc test (for multiple group comparisons). MK expression at different time points during preadipocyte differentiation was compared using repeated measures of ANOVA. Pearson’s test was used for the correlation analyses in the clinical study. All statistical analyses were performed with SPSS 13.0 (Chicago, IL). *P*<0.05 was considered statistically significant.

## Results

### MK Expression is Dynamically Regulated during Preadipocyte Differentiation

**Figure 1:**
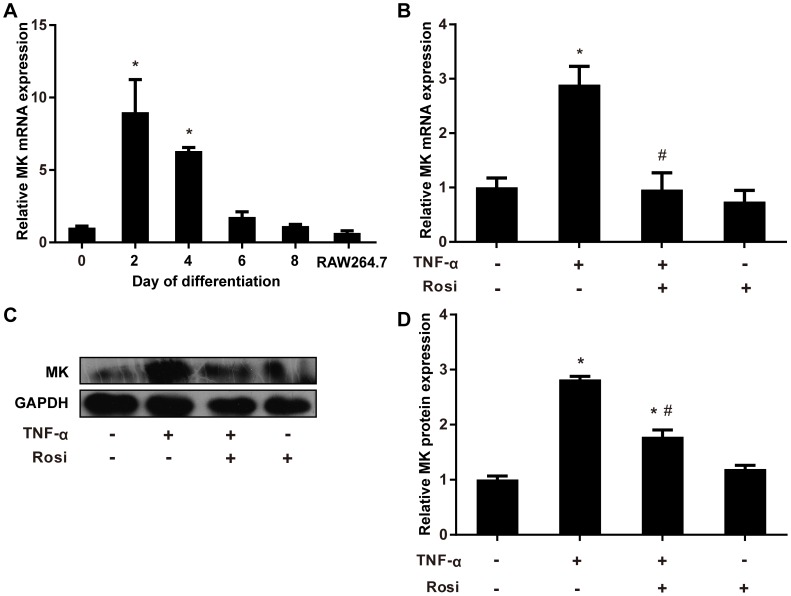
MK expression during preadipocyte differentiation and its regulation by inflammatory modulators in mature adipocytes. **A.** 3T3-L1 preadipocytes were exposed to differentiation medium and MK mRNA expression was evaluated by quantitative RT-PCR at the indicated time points. Additionally, MK expression in RAW264.7 macrophages was also examined. Relative MK mRNA expression was expressed as fold of the D0 value. **B**, **C.** Differentiated 3T3-L1 adipocytes were incubated with or without TNF-α for 24 h in the presence or not of rosiglitazone (Rosi). MK expression was evaluated by quantitative RT-PCR (B) or western blot (C), and was expressed as fold of controls. D, Intensity of bands was quantified by densitometry. MK levels were normalized to GAPDH and expressed as fold of controls. Data are mean ± SE; n = 3. *P<0.05 versus control cells, #*P*<0.05 versus cells only treated with TNF-α.

To explore the role of MK in adipocytes, we first assessed the expression pattern of MK upon 3T3-L1 preadipocyte differentiation. As previously reported [21], MK mRNA expression increased dramatically after differentiation and reached a peak on D2 (9-fold relative to D0, *P*<0.05) (Figure 1A). Thereafter, the expression of MK gradually decreased and returned to the D0 levels on D8 (Figure 1A), consistent with its mitogenic effect on preadipocytes after initiation of differentiation [21]. Additionally, MK mRNA expression levels in differentiated 3T3-L1 adipocytes on D8 were comparable to those in RAW264.7 macrophages (Figure 1A).

### TNF-α and Rosiglitazone Regulate MK Expression in Adipocytes

It has been reported that MK is upregulated by inflammatory stimuli (e.g., TNF-α and IL-1β) in several cell types [26], [27]. To determine whether MK expression in adipocytes is also modulated by inflammatory modulators, we treated 3T3-L1 adipocytes with TNF-α and/or rosiglitazone, which are well known to promote and attenuate inflammation in adipocytes, respectively [28], [29]. As shown in Figure 1B, TNF-α treatment led to a marked increase in MK mRNA expression in 3T3-L1 adipocytes. Of note, this increase was completely abrogated by rosiglitazone (Figure 1B). Consistent with the mRNA results, TNF-α induced MK protein expression in adipocytes, which was significantly attenuated by rosiglitazone (Figure 1C and D). Together, MK expression in adipocytes seems to be regulated by inflammatory modulators.

### MK Expression is Increased in Adipose Tissue of Obese ob/ob Mice

**Figure 2:**
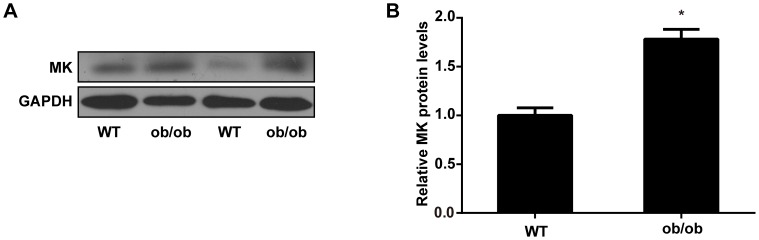
MK expression is increased in epididymal adipose tissue of obese ob/ob mice. **A**. Western blot analysis of MK protein levels in epididymal adipose tissue of leptin deficiency mice (ob/ob) and their wild-type littermate controls (WT) (n = 4 per group). Representative results are shown. **B.** Intensity of bands was quantified by densitometry. MK protein levels were normalized to GAPDH protein levels and expressed as fold of controls. Data are mean ± SE. *P<0.05 versus controls.

**Figure 3:**
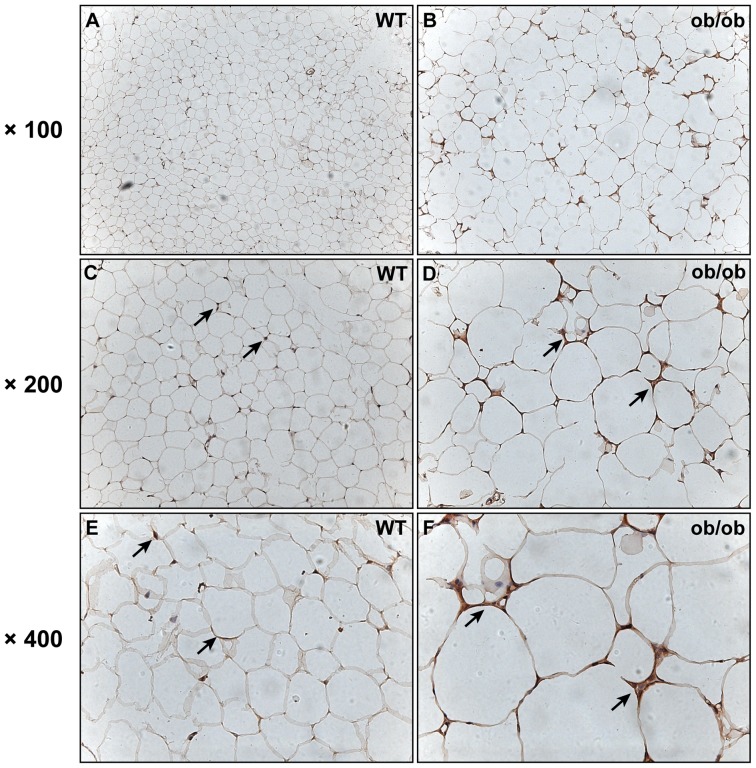
Immunohistochemical analysis of MK expression in epididymal adipose tissue of mice. MK expression was analyzed by immunohistochemistry in epididymal adipose tissue of leptin deficiency mice (ob/ob) (B, D, F) and their wild-type littermate controls (WT) (A, C, E) (n = 4 per group). Representative results are shown. Arrow, positive staining.

To probe the role of MK in vivo, we then examined its expression levels in epididymal adipose tissue of ob/ob mice, a well-characterized model of severe genetic obesity and insulin resistance due to leptin deficiency [30]. As assessed by western blot analysis, MK protein expression was significantly increased in epididymal adipose tissue of ob/ob mice when compared with their lean littermate controls (Figure 2). In addition, we performed immunohistochemical analysis of adipose tissue from mice. As shown in Figure 3, MK was detected in both adipocytes and stromal cells of adipose tissue. Moreover, MK expression was increased in epididymal adipose tissue of ob/ob mice relative to controls (Figure 3). Together, these results indicate an association between MK and obesity in vivo.

### Serum MK Levels are Associated with Obesity in Humans

**Figure 4:**
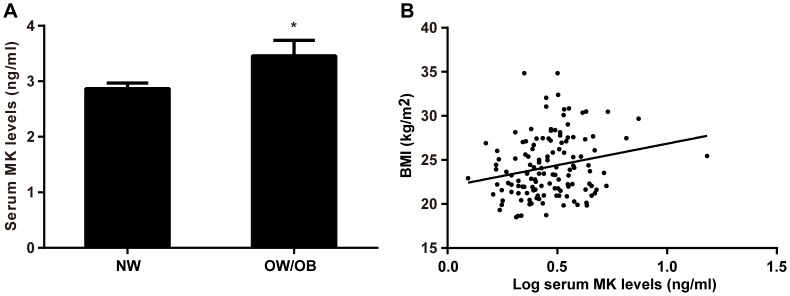
Serum levels of MK are associated with obesity in humans. **A**. Serum levels of MK in normal weight (n = 84) and overweight/obese subjects (n = 51). Data are means ± SE. NW, normal weight; OW/OB, overweight/obese. **B**. Correlation between serum levels of MK (Log transformed) and BMI. *P<0.05 versus normal weight subjects.

In light of the above in vitro and animal results, we further assessed the clinical relevance of MK in humans by determining its serum levels in overweight/obese subjects. Clinical and biochemical characteristics of the study subjects are shown in Table 2. Serum MK levels were significantly higher in overweight/obese subjects than in control subjects, [3.46±0.28 ng/ml versus 2.87±0.10 ng/ml, *P* = 0.022] (Figure 4A). After adjustment for age and sex by covariance analysis, the difference remained significant [3.50±0.20 ng/ml versus 2.84±0.16 ng/ml, *P* = 0.012]. Furthermore, there was a positive correlation between serum MK levels and BMI (*r* = 0.214, *P* = 0.013, Figure 4B), and the correlation remained significant after adjusting for age and sex by partial correlation analysis (*r* = 0.234, *P* = 0.007). Together, our results show that MK is associated with obesity in humans.

**Table 2:** Clinical and biochemical characteristics of the study subjects.

Characteristics	NormalWeight	Overweight/obese	*P* Value
Number ofSubjects	84	51	
Age (years)	51.1±1.6	49.4±1.8	0.093
Male, n (%)	32 (38)	27 (53)	0.092
BMI (kg/m^2^)	22.0±0.2	27.9±0.3	<0.001
SBP (mmHg)	119.6±1.9	127.8±2.3	0.007
DBP (mmHg)	74.7±0.9	80.0±1.2	0.001
FBG (mmol/L)	4.74±0.04	4.94±0.05	0.004
TG (mmol/L)	1.31±0.08	1.86±0.12	<0.001
TC (mmol/L)	4.92±0.09	5.03±0.14	0.495
LDL-C (mmol/L)	3.14±0.09	3.29±0.13	0.338
HDL-C (mmol/L)	1.50±0.04	1.30±0.05	0.001

Data are presented as number (percentage) for categorical data, mean ± SE for continuous data. BMI, body mass index; SBP, systolic blood pressure; DBP, diastolic blood pressure; FBG, fasting blood glucose; TG, triglycerides; TC, total cholesterol; LDL-C; low-density lipoprotein cholesterol; HDL-C; high-density lipoprotein cholesterol.

### MK Impairs Insulin Signaling in 3T3-L1 Adipocytes

**Figure 5:**
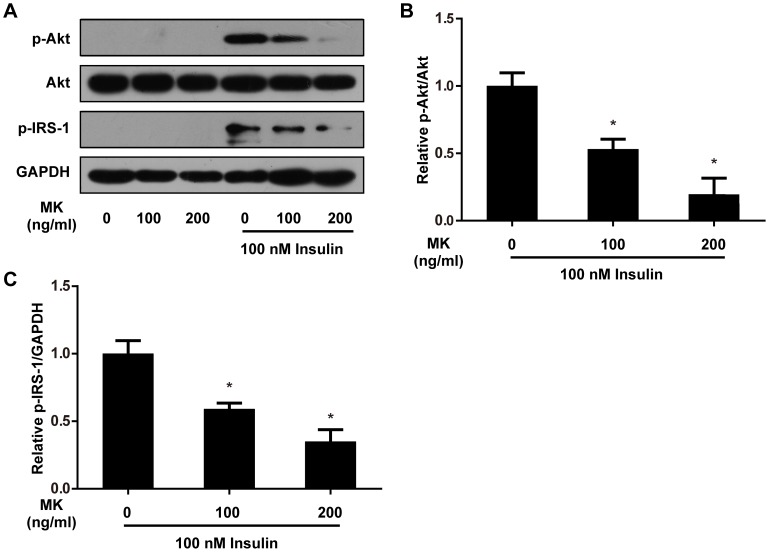
MK inhibits insulin signaling in 3T3-L1 adipocytes. **A.** Differentiated 3T3-L1 adipocytes were treated with recombinant mouse MK or vehicle for 24 h, and subsequently stimulated with insulin for 10 min. Phosphorylation of Akt (Ser473) and IRS-1 (Tyr612) were assessed by western blot analysis and representative results are shown. **B, C.** Intensity of bands was quantified by densitometry. Phosphorylated protein levels were normalized to total protein or GAPDH and expressed as fold of insulin-stimulated controls. Data are mean ± SE; n = 3. *P<0.05 versus control cells stimulated with insulin.

We next sought to explore the pathophysiological significance of increased MK expression in obesity. Given its proinflammatory properties, we determined whether MK could attenuate insulin signaling in adipocytes, just like other inflammatory mediators [7]. Fully differentiated 3T3-L1 adipocytes were exposed to recombinant MK for 24 h and insulin signal transduction was then examined. As shown in Figure 5A and B, insulin-stimulated phosphorylation of Akt on Ser473, a commonly used marker of insulin signaling [31], was markedly reduced by MK treatment. Additionally, insulin signaling event upstream of Akt was also assessed. Consistently, MK decreased specific tyrosine phosphorylation of insulin receptor substrate-1 (IRS-1) on Tyr612 in response to insulin stimulation (Figure 5A and C). Taken together, our results show that MK impairs insulin signaling in 3T3-L1 adipocytes.

### MK Reduces Insulin-stimulated GLUT4 Translocation in 3T3-L1 Adipocytes

**Figure 6:**
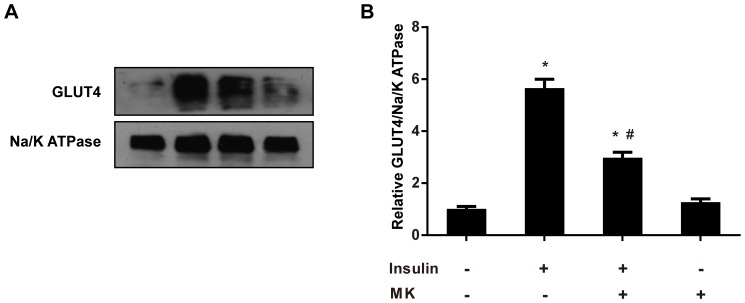
MK inhibits insulin-stimulated GLUT4 translocation in 3T3-L1 adipocytes. **A.** Differentiated 3T3-L1 adipocytes were treated with recombinant mouse MK or vehicle for 24 h, and subsequently stimulated with insulin for 30 min. Plasma membrane proteins were isolated and GLUT4 was assessed by western blot analysis. Representative results are shown. **B.** Intensity of bands was quantified by densitometry. GLUT4 protein levels were normalized to Na/K ATPase and expressed as fold of controls. Data are mean ± SE; n = 3. *P<0.05 versus control cells. #*P*<0.05 versus cells only treated with insulin.

Insulin-stimulated translocation of the glucose transporter GLUT4 to the cell surface in adipocytes is the basis for insulin-stimulated glucose uptake. In light of the inhibitory effects of MK on insulin signaling, we tested whether MK could reduce insulin-stimulated translocation of GLUT4. After 24 h treatment with MK, 3T3-L1 adipocytes were stimulated with 100 nM insulin for 30 min and plasma membrane GLUT4 protein was evaluated by western blot analysis. As shown in Figure 6, MK significantly reduced insulin-stimulated translocation of GLUT4 to the plasma membrane, consistent with its inhibitory effects on insulin signaling in adipocytes.

### MK Does Not Activate NFκB Signaling in Adipocytes

**Figure 7:**
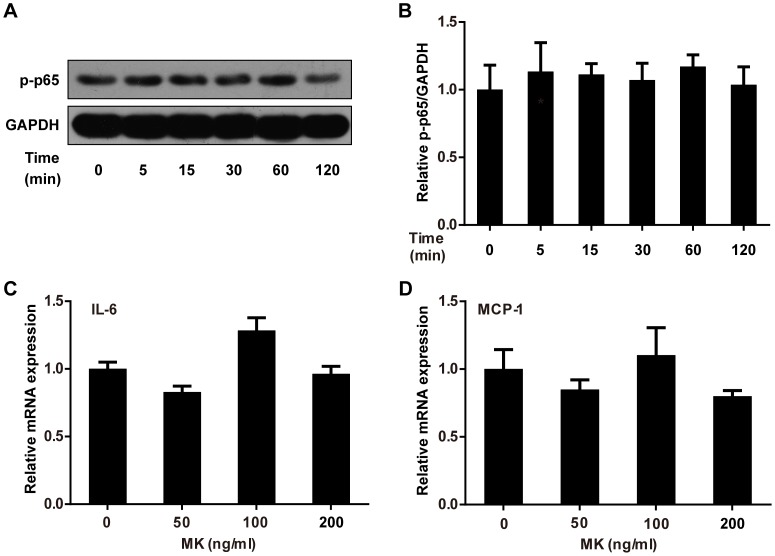
MK does not activate NFκB signaling in 3T3-L1 adipocytes. **A.** Differentiated 3T3-L1 adipocytes were treated with recombinant MK for the indicated time periods. Phosphorylation of p65/NFκB (Ser536) was assessed by western blot analysis and representative results are shown. **B.** Intensity of bands was quantified by densitometry. Phosphorylated p65/NFκB levels were normalized to GAPDH and expressed as fold of controls. **C, D.** Differentiated 3T3-L1 adipocytes were treated with increasing dose of recombinant MK for 16 h. IL-6 (C) and MCP-1 (D) mRNA expression were evaluated by quantitative RT-PCR. Relative gene expression was expressed as fold of controls. Data are mean ± SE; n = 3. *P<0.05 versus controls.

The possible mechanisms underlying the suppressive effects of MK on insulin signaling were further investigated. As NFκB signaling plays a central role in insulin resistance and can be activated by MK in other cell types [32], [33], we examined the actions of MK on this pathway in adipocytes. 3T3-L1 adipocytes were treated with recombinant MK, and the phosphorylaion of NFκB as well as the expression of inflammatory mediators was assessed. As shown in Figure 7A and B, MK did not induce the phosphorylation of p65/NFκB. Accordingly, IL-6 and MCP-1 mRNA expression in adipocytes were unchanged by MK (Figure 7C and D). Thus, MK does not activate NFκB signaling in adipocytes.

### MK Activates the STAT3-SOCS3 Pathway in Adipocytes

**Figure 8:**
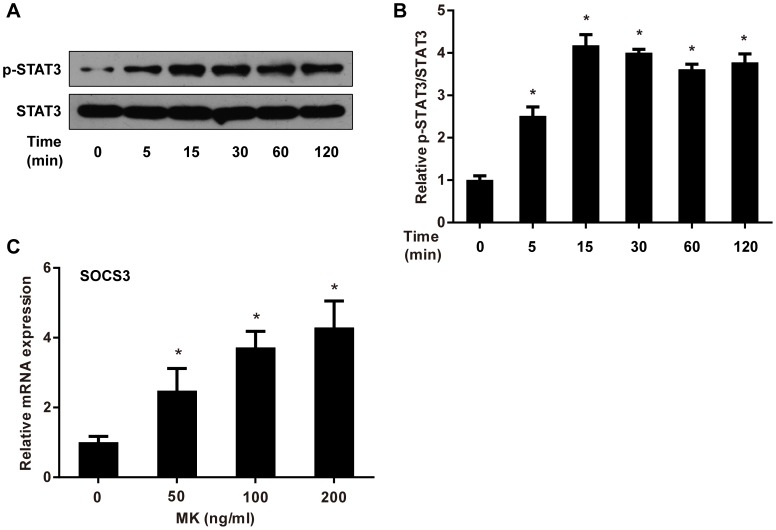
MK activates the STAT3-SOCS3 pathway in 3T3-L1 adipocytes. **A.** Differentiated 3T3-L1 adipocytes were treated with recombinant MK for the indicated time periods. Phosphorylated (Tyr705) and total STAT3 protein were assessed by western blot analysis and representative results are shown. **B.** Intensity of bands was quantified by densitometry. Phosphorylated STAT3 levels were normalized to total STAT3 and expressed as fold of controls. **C.** Differentiated 3T3-L1 adipocytes were treated with increasing dose of recombinant mouse MK for 16 h. SOCS3 mRNA expression was evaluated by quantitative RT-PCR. Relative gene expression was expressed as fold of controls. Data are mean ± SE; n = 3. *P<0.05 versus controls.

In addition to NFκB signaling, the STAT3-SOCS3 pathway is critical in cytokine-induced insulin resistance [34]–[36]. Of note, MK has been reported to activate STAT3 in 3T3-L1 preadipocytes, which prompted us to test whether MK also stimulates this signaling pathway in mature adipocytes. As shown in Figure 8A and B, as early as 5 min after MK treatment, significantly increased phosphorylation of STAT3 on Tyr705 was observed. This increase sustained up to 120 min. Moreover, the expression of SOCS3, a downstream target of STAT3, was also induced by MK in a dose-dependent manner (Figure 8C). These findings indicate that MK is a potent activator of the STAT3-SOCS3 pathway in mature adipocytes.

## Discussion

As a novel endocrine and immune organ, adipose tissue secretes a variety of adipokines that are directly involved in inflammation and insulin resistance. In this study, we investigated the association of MK with obesity and its actions on adipocytes. MK was found to be expressed in adipocytes and regulated by inflammatory modulators. Notably, MK levels were increased in adipose tissue of obese mice and in serum of overweight/obese subjects as compared with their controls. In vitro experiments further revealed inhibitory effects of MK on insulin signaling in 3T3-L1 adipocytes, with activation of the STAT3-SOCS3 pathway. Our findings suggest a potential role of MK in obesity-induced insulin resistance.

MK is expressed in multiple cell types, including various immune and cancer cells [37], [38]. Here, we found MK expression in both 3T3-L1 preadipocytes and mature adipocytes. In preadipocytes, MK expression increased immediately after differentiation and then declined progressively to the beginning levels, consistent with its essential role in promoting the mitotic clonal expansion of preadipocytes [21]. In mature adipocytes, MK was regulated by inflammatory modulators. TNF-α treatment led to a marked increase in MK expression, which was completely abolished by rosiglitazone, a potent PPARγ agonist with antiinflammatory actions. Thus, in line with its inflammatory properties, MK seems closely associated with the inflammatory state of mature adipocytes.

In addition to the adipocyte cell line in vitro, MK is also expressed in adipose tissue of mice. Importantly, MK expression was upregulated in epididymal adipose tissue of obese mice. Furthermore, overweight/obese humans had significantly increased serum MK levels compared with control subjects, with a positive correlation between serum MK and BMI. Collectively, MK is associated with obesity in both mice and humans. The mechanisms for MK upregulation in obese adipose tissue may be multiple and remain to be elucidated. TNF-α, which is increased in obesity [39], induces MK expression in adipocytes, and is therefore a potential candidate for the upregulation of MK. As MK is also expressed by macrophages, which are recruited into adipose tissue in obesity, they may be another source of MK in adipose tissue. In fact, we observed increased expression of MK in stromal cells, which are largely composed of macrophages, in adipose tissue of ob/ob mice compared with controls. Nevertheless, the relative contribution of adipocytes and macrophages to the elevated expression of MK in obese adipose tissue remains to be determined. In addition, as a secreted protein by adipose tissue, MK serum concentration in mice and its relationship with obesity warrant future study.

Adipose tissue produces a range of adipokines that are directly involved in insulin resistance [7]. Herein, we showed that MK suppressed insulin signaling in adipocytes, as indicated by reduced phosphorylation of Akt and IRS-1 in response to insulin stimulation. These findings provide the first evidence that MK may be a novel inducer of insulin resistance. Since MK expression was increased in adipose tissue of obese mice, it warrants further investigation whether MK induces insulin resistance in vivo. Moreover, as serum MK levels were significantly elevated in obese subjects and correlated with BMI, further analysis of its relationship with insulin sensitivity will provide additional evidence for its actions on insulin resistance in humans. In addition, given the chemotactic activities of MK towards macrophages [16], which play a central role in obesity-induced inflammation and insulin resistance [40], future studies will also investigate the involvement of MK in macrophage recruitment into adipose tissue during obesity.

Though MK attenuates insulin signaling in adipocytes, the signal events by which MK interacts with insulin signal transduction remain to be clarified. The STAT3-SOCS3 pathway has been demonstrated to play a critical role in insulin resistance [34], [41]. On activation, STAT3 dimerizes and translocates to the nucleus, inducing the expression of SOCS3, which in turn inhibits insulin signaling by direct interaction with the insulin receptor and by preventing the coupling of IRS-1 with the insulin receptor [42], [43]. To date, a range of adipokines (e.g., IL-6, TNF-α and resistin) have been reported to promote insulin resistance in adipocytes through the STAT3-SOCS3 pathway [34]–[36]. In this study, we observed that MK also activated the STAT3-SOCS3 pathway in 3T3-L1 adipocytes, consistent with previous studies showing stimulative effects of MK on STAT3 in preadipocytes and keratinocytes [21], [44]. Thus, MK is a potent activator of the STAT3-SOCS3 signaling cascade, which may mediate the inhibitory effects of MK on insulin signaling in adipocytes.

**Figure 9:**
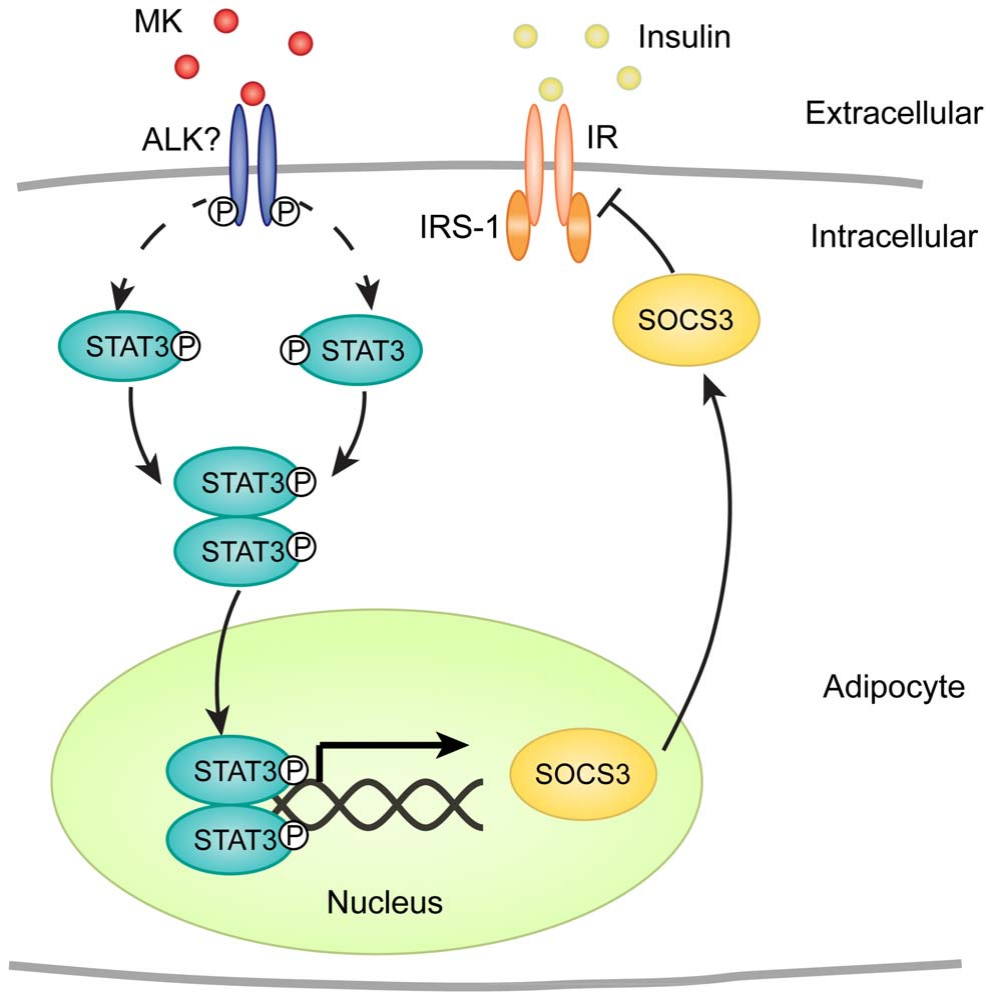
Schematic diagram of mechanisms of MK actions in adipocytes. Probably through anaplastic lymphoma kinase (ALK), a transmembrane receptor, MK induces tyrosine phosphorylation and dimeration of STAT3, which translocates to the nucleus and stimulates the transcription of SOCS3. Subsequently, SOCS3 inhibits insulin signaling by interacting with insulin receptor and IRS-1.

Another question not addressed is how MK activates the STAT3-SOCS3 pathway in adipocytes. Previous studies have proposed multiple molecules as the receptor of MK, including anaplastic lymphoma kinase (ALK), protein-tyrosine phosphatase ζ (PTPζ), low density lipoprotein receptor-related protein (LRP) and integrin [8], [45]–[47]. Among them, ALK is a transmembrane receptor tyrosine kinase that has been shown to activate STAT3 [48]. Additionally, we detected ALK expression in adipocytes (data not shown). Thus, it may be through ALK that MK activates the STAT3-SOCS3 pathway in adipocytes, which further impairs insulin signal transduction, as illustrated in Figure 9.

In summary, we show here that MK is expressed in adipocytes and is associated with obesity in both mice and humans. Moreover, as revealed by reduced phosphorylation of Akt and IRS-1 and decreased GLUT4 translocation in response to insulin stimulation, MK suppresses insulin signaling in adipocytes, associated with activation of the STAT3-SOCS3 signaling pathway. Therefore, MK is a potential link between obesity and insulin resistance, and may offer a new target to treat insulin resistance and other obesity-associated diseases.
